# Linear dynamics of classical spin as Möbius transformation

**DOI:** 10.1038/s41598-017-01326-x

**Published:** 2017-04-26

**Authors:** Alexey Galda, Valerii М. Vinokur

**Affiliations:** 1 James Franck Institute, University of Chicago, Chicago, Illinois 60637 USA; 2 Materials Science Division, Argonne National Laboratory, Argonne, Illinois 60439 USA; 3 Computation Institute, University of Chicago, 5735 S. Ellis Avenue, Chicago, Illinois 60637 USA

## Abstract

Though the overwhelming majority of natural processes occur far from the equilibrium, general theoretical approaches to non-equilibrium phase transitions remain scarce. Recent breakthroughs introduced a description of open dissipative systems in terms of non-Hermitian quantum mechanics enabling the identification of a class of non-equilibrium phase transitions associated with the loss of combined parity (reflection) and time-reversal symmetries. Here we report that the time evolution of a single classical spin (e.g. monodomain ferromagnet) governed by the Landau-Lifshitz-Gilbert-Slonczewski equation in the absence of magnetic anisotropy terms is described by a Möbius transformation in complex stereographic coordinates. We identify the *parity-time* symmetry-breaking phase transition occurring in spin-transfer torque-driven linear spin systems as a transition between hyperbolic and loxodromic classes of Möbius transformations, with the critical point of the transition corresponding to the parabolic transformation. This establishes the understanding of non-equilibrium phase transitions as topological transitions in configuration space.

## Introduction

The interest to dissipative spin-transfer torque (STT)-driven dynamics of a spin, described by Landau-Lifshitz-Gilbert-Slonczewski (LLGS) equation^1–3^ is two-fold. On the application side, spin controlled by the applied spin-polarized current is an elemental unit for a wealth of spintronic applications^4–7^. On the fundamental side, complete quantitative understanding of single-spin dynamics provides the essential tool for predictive description of many complex spin systems.

Analytical studies of nonlinear spin dynamics in nanomagnetic devices and structures have been the focus of active research for many decades (see, e.g., refs 8, 9 and references therein). It has recently been shown that nonequilibrium classical spin dynamics described by the LLGS equation naturally follows from the non-Hermitian extension of Hamiltonian formalism^10^. Within this framework, the nonconservative effects of Gilbert damping and applied Slonczewski STT^3^ originate from the imaginary part of the system’s Hamiltonian. This new technique has enabled important advances in the field of nonlinear spin dynamics, including the discovery of *parity-time* (${\mathscr{P}}{\mathscr{T}}$) symmetry-breaking in systems with mutually orthogonal applied magnetic field and STT. This new type of phase transitions in spin systems is possible due to the invariance of STT action under simultaneous operations of time-reversal and reflection with respect to the direction of spin polarization.

Here we find that the ${\mathscr{P}}{\mathscr{T}}$ symmetry-breaking phase transition occurring in STT-driven linear spin systems (i.e. the systems designed to have zero or negligibly small magnetic anisotropy) is a transition between hyperbolic and loxodromic classes of Möbius transformations governing the spin dynamics. The critical point of the phase transition corresponds to the merging of two fixed points of these Möbius transformations (equilibrium points of spin dynamics) into a single fixed point of a parabolic transformation. This establishes that non-equilibrium phase transitions associated with ${\mathscr{P}}{\mathscr{T}}$ symmetry-breaking are topological transitions in configuration space.

We undertake the analytical study of dissipative STT-driven dynamics of a single classical spin described by a linear (in spin operators) non-Hermitian Hamiltonian. We show that the combined effect of an external magnetic field, Gilbert damping, and applied Slonczewski STT can be incorporated in the effective action of a *complex* magnetic field. We derive an equation of motion in complex stereographic coordinates that assumes the form of a Riccati equation. This allows a recasting of the equation of motion into linear form without any approximations beyond the initial choice of the non-Hermitian spin Hamiltonian. The equation of motion in stereographic projection coordinates admits an exact solution in the form of a Möbius transformation of ${{\mathbb{C}}}^{2}$. The correspondence between different regimes of spin dynamics and classes of Möbius transformations is established and illustrated on the example of the ${\mathscr{P}}{\mathscr{T}}$ symmetry-breaking phenomenon, which is identified as a transition between elliptic and loxodromic Möbius transformations via a parabolic transformation.

The equation of motion can also be recast into the linear form by employing complex homogeneous coordinates. The linear form of the spin dynamics equation provides a solid foundation for the study of nonlinear effects in single and coupled spin systems, including chaotic dynamics^11, 12^, spin-wave instabilities^13^, and solitons^14^.

## Results and Discussion

We study the most general *linear* version of the spin Hamiltonian proposed by Galda and Vinokur^10^,

1 $$\hat{{\mathscr{H}}}=(\frac{\gamma {\bf{H}}+i{\bf{j}}}{1-i\alpha })\cdot \hat{{\bf{S}}},$$

where **H** is the applied magnetic field, the imaginary field *i* **j** is responsible for the action of STT, and the phenomenological constant *α* describes Gilbert damping. The corresponding LLGS equation of spin dynamics reads

2 $$\dot{{\bf{S}}}=\gamma {\bf{H}}\times {\bf{S}}+\frac{\alpha }{S}\dot{{\bf{S}}}\times {\bf{S}}+\frac{1}{S}[{\bf{j}}\times {\bf{S}}]\times {\bf{S}},$$

where *γ* = *gμ*_B_/*ħ* is the absolute value of the gyromagnetic ratio, $g\simeq 2$, and *S* ≡ |**S**| is the total spin (constant in time). The first two terms in Eq. (2) describe the standard Landau-Lifshitz (LL) torque and dissipation in Gilbert form, while the last one is responsible for Slonczewski STT.

To show that Hamiltonian (1) yields the above LLGS dynamics equation in the classical limit (*S* → ∞), it is most convenient to consider *SU*(2) spin-coherent states^15, 16^ $|\zeta \rangle ={{\rm{e}}}^{\zeta {\hat{S}}_{+}}|S,-S\rangle$, where ${\hat{S}}_{\pm }={\hat{S}}_{x}\pm i{\hat{S}}_{y}$, and $\zeta \in {\mathbb{C}}$ is the standard stereographic projection of the spin direction on a unit sphere, $\zeta =({s}_{x}+i{s}_{y}\mathrm{)/(1}-{s}_{z})$, with the south pole (spin-down state) corresponding to *ζ* = 0.

The Hamiltonian function in spin-coherent states reads

3 $$ {\mathcal H} (\zeta ,\bar{\zeta })=\frac{\langle \zeta |\hat{ {\mathcal H} }|\zeta \rangle }{\langle \zeta |\zeta \rangle },$$

which gives^10^ the following compact form of Hamilton’s equation of motion for classical spin:

4 $$\dot{\zeta }=i\frac{{(1+|\zeta {|}^{2})}^{2}}{2S}\frac{\partial  {\mathcal H} }{\partial \bar{\zeta }},$$

where the factor ${(1+|\zeta {|}^{2})}^{2}/2S$ ensures invariance of measure on a two-sphere. Let us now normalize and rewrite the linear non-Hermitian Hamiltonian (1) in terms of dimensionless variables:

5 $${\hat{{\mathscr{H}}}}_{0}\equiv \hat{{\mathscr{H}}}/S=\mathop{{\bf{h}}}\limits^{\sim}\cdot \hat{{\bf{s}}},$$

where **s** ≡ **S**/S, and the effects of the applied magnetic field, Gilbert damping and Slonczewski STT contributions are all incorporated into the complex magnetic field $\mathop{{\bf{h}}}\limits^{\sim}=({\mathop{h}\limits^{\sim}}_{x},{\mathop{h}\limits^{\sim}}_{y},{\mathop{h}\limits^{\sim}}_{z})\in {\mathbb{C}}$. The equation of motion (4) for the linear classical spin Hamiltonian (5) can be rewritten as a linear matrix ordinary differential equation:

6 $$\frac{d}{dt}[\begin{matrix}\xi (t)\\ \eta (t)\end{matrix}]=A[\begin{matrix}\xi (t)\\ \eta (t)\end{matrix}],$$

7 $$A=\frac{i}{2}\sum _{k=x,y,z}{\tilde{h}}_{k}{\sigma }_{k},$$

where *σ*_*k*_ are Pauli matrices, and $\zeta (t)\equiv \xi (t)/\eta (t)$. The pair of complex functions $\{\xi ,\eta \}$ are called homogeneous coordinates of *ζ* ^17^, such that each ordered pair {*ξ*, *η*} (except {0, 0}) corresponds to a unique stereographic projection coordinate *ζ*. The initial conditions for Eq. (6) can be chosen as $\xi \mathrm{(0)}=\zeta \mathrm{(0),}\,\eta \mathrm{(0)}=1$.

The solution in terms of stereographic projection coordinates *ζ* takes the simple form of a Möbius transformation:

8 $$\zeta (t)=\frac{{M}_{11}\zeta \mathrm{(0)}+{M}_{12}}{{M}_{21}\zeta \mathrm{(0)}+{M}_{22}}\equiv M[\zeta \mathrm{(0)}],$$

where the normalized (det *M* = 1) transformation matrix is given by the matrix exponential:

9 $$M={{\rm{e}}}^{At}.$$

It is important that non-conservative spin dynamics only takes the form of a Möbius transformation for systems described by linear spin Hamiltonians. Experimentally this corresponds to systems designed to have negligibly small magnetic anisotropies. The inclusion of nonlinear anisotropy terms in the spin Hamiltonian^10^ inevitably leads to other types of spin dynamics equations due to the action of spin-orientation-dependent effective magnetic fields on the spin. The equation of motion (6) illustrates that the classical spin dynamics discussed here can be written in linear form despite the nonlinear nature of the LLGS Eq. (2) it reproduces. Understanding this linear system and its solutions represents a crucial step in describing nonlinear STT-driven magnetic systems.

### Möbius transformation

We now study the solution of Eq. (4) for linear spin Hamiltonians. Without loss of generality, we can take ${\tilde{h}}_{z}=0$ and ${\rm{Im}}\,({\tilde{h}}_{x})=0$ in Eq. (5) by choosing the *z* axis along $[{\rm{R}}{\rm{e}}(\mathop{{\bf{h}}}\limits^{\sim})\times {\rm{I}}{\rm{m}}(\mathop{{\bf{h}}}\limits^{\sim})]$ and *y* axis along ${\rm{I}}{\rm{m}}(\mathop{{\bf{h}}}\limits^{\sim})$, while ${h}_{x}\equiv {\rm{Re}}\,({\tilde{h}}_{x})$ and ${\tilde{h}}_{y}\in {\mathbb{C}}$ can be arbitrary:

10 $${\hat{ {\mathcal H} }}_{0}={h}_{x}{\hat{s}}_{x}+{\tilde{h}}_{y}{\hat{s}}_{y}.$$

The equation of motion for this Hamiltonian takes the form of a Riccati equation:

11 $$\dot{\zeta }(t)=-i\frac{{h}_{x}-i{\mathop{h}\limits^{\sim}}_{y}}{2}[{\zeta }^{2}(t)-\frac{{h}_{x}+i{\mathop{h}\limits^{\sim}}_{y}}{{h}_{x}-i{\mathop{h}\limits^{\sim}}_{y}}]$$

with two fixed points,

12 $${\zeta }_{\mathrm{1,2}}=\pm \sqrt{\frac{{h}_{x}+i{\tilde{h}}_{y}}{{h}_{x}-i{\tilde{h}}_{y}}},$$

and the solution

13 $$\zeta (t)=\frac{\cos (\frac{\sqrt{{h}_{x}^{2}+{\mathop{h}\limits^{\sim}}_{y}^{2}}}{2}t)\zeta (0)+\frac{i{h}_{x}+{\mathop{h}\limits^{\sim}}_{y}}{\sqrt{{h}_{x}^{2}+{\mathop{h}\limits^{\sim}}_{y}^{2}}}\sin (\frac{\sqrt{{h}_{x}^{2}+{\mathop{h}\limits^{\sim}}_{y}^{2}}}{2}t)}{\frac{i{h}_{x}-{\mathop{h}\limits^{\sim}}_{y}}{\sqrt{{h}_{x}^{2}+{\mathop{h}\limits^{\sim}}_{y}^{2}}}\sin (\frac{\sqrt{{h}_{x}^{2}+{\mathop{h}\limits^{\sim}}_{y}^{2}}}{2}t)\zeta (0)+\cos (\frac{\sqrt{{h}_{x}^{2}+{\mathop{h}\limits^{\sim}}_{y}^{2}}}{2}t)}.$$

Equation (13) shows that the time evolution of a classical spin generated by an arbitrary linear non-Hermitian Hamiltonian presented in stereographic projection coordinates, is nothing but a Möbius transformation of ${{\mathbb{C}}}^{2}$:

14 $$\begin{matrix}[M]=[{{\rm{e}}}^{\frac{i}{2}({h}_{x}{\sigma }_{x}+{\mathop{h}\limits^{\sim}}_{y}{\sigma }_{y})t}]\\ \quad =[\begin{matrix}\cos (\frac{\sqrt{{h}_{x}^{2}+{\mathop{h}\limits^{\sim}}_{y}^{2}}}{2}t) & \frac{i{h}_{x}+{\mathop{h}\limits^{\sim}}_{y}}{\sqrt{{h}_{x}^{2}+{\mathop{h}\limits^{\sim}}_{y}^{2}}}\sin (\frac{\sqrt{{h}_{x}^{2}+{\mathop{h}\limits^{\sim}}_{y}^{2}}}{2}t)\\ \frac{i{h}_{x}-{\mathop{h}\limits^{\sim}}_{y}}{\sqrt{{h}_{x}^{2}+{\mathop{h}\limits^{\sim}}_{y}^{2}}}\sin (\frac{\sqrt{{h}_{x}^{2}+{\mathop{h}\limits^{\sim}}_{y}^{2}}}{2}t) & \cos (\frac{\sqrt{{h}_{x}^{2}+{\mathop{h}\limits^{\sim}}_{y}^{2}}}{2}t)\end{matrix}],\end{matrix}$$

in accordance with Eqs. (7), (9) and (10).

### Classification of Möbius transformations based on spin dynamics

**Figure 1:**
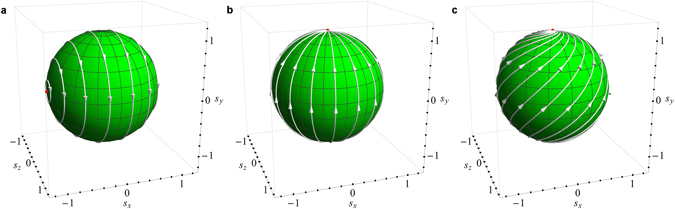
Geodesics of an elliptic (**a**), hyperbolic (**b**), and loxodromic (**c**) Möbius transformation, corresponding in spin dynamics to the applied real magnetic field along the *x* axis (**a**), imaginary magnetic field along the *y* axis (**b**), and complex magnetic field along the *y* axis (**c**).

The traditional classification of Möbius transformations based on the number and type of fixed points distinguishes three different classes: elliptic, loxodromic (including hyperbolic as a special case) and parabolic transformations, which can be identified by calculating tr^2^ *M* ^17^. Here we show that all Möbius transformations can be obtained from a superposition of only two basic transformations, elliptic and hyperbolic, because in spin dynamics these two directly correspond to applied real and imaginary magnetic fields. An elliptic Möbius transformation induces a uniform rotation of the entire Riemann sphere around a central axis, while a hyperbolic transformation produces antipodal expansion and contraction centers, see Fig. 1, where the lines depict invariant geodesics of the corresponding Möbius transformation on the sphere. According to this consideration, every elliptic and hyperbolic transformation is fully determined by two parameters: ‘direction’ and ‘amplitude’. Together, these parameters define the direction of geodesics, including the location of the fixed points and the displacement of points on the Riemann sphere along geodesics upon the transformation. In these terms, the action of a real magnetic field ${\bf{h}}=({h}_{x},{h}_{y},{h}_{z})$ leads to spin dynamics governed by an elliptic Möbius transformation with the normalized transformation matrix $M=\exp (\frac{i}{2}{\sum }_{k=x,y,z}{h}_{k}{\sigma }_{k})$. Similarly, an imaginary applied magnetic field produces spin dynamics associated with a hyperbolic transformation, with the matrix of the transformation containing purely imaginary coefficients *h*_*k*_ in the exponent. Given that any complex matrix *M*, such that det *M* = 1, can be uniquely represented as a matrix exponential of the form $M=\exp (\frac{i}{2}{\sum }_{k=x,y,z}{\mathop{h}\limits^{\sim}}_{k}{\sigma }_{k})$, where ${\tilde{h}}_{k}\in {\mathbb{C}}$, it follows that any Möbius transformation is a superposition of an elliptic and a hyperbolic transformations.

**Figure 2:**
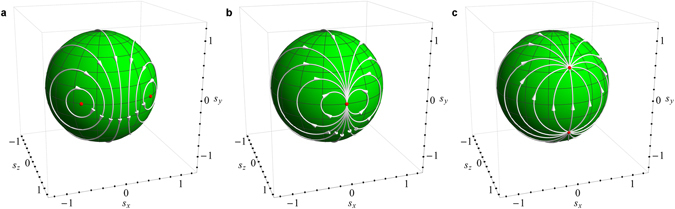
Transition between elliptic (**a**) and loxodromic (**c**) Möbius transformations by increasing *ε* past the critical value 1, at which one obtains the parabolic transformation (**b**). In classical spin dynamics this transition corresponds to ${\mathscr{P}}{\mathscr{T}}$ symmetry-breaking.

A general loxodromic transformation has two fixed points, an attractive and repulsive nodes, which in spin dynamics correspond to the stable and unstable equilibrium states. The transformation (14) is loxodromic when both of the following two conditions are met: (a) $\beta \equiv \,{\rm{Im}}\,{\tilde{h}}_{y}\ne 0$ and (b) ${h}_{y}\equiv {\rm{Re}}\,{\tilde{h}}_{y}\ne 0$ if *h*_*x*_ ≠ 0, which follows from the condition ${{\rm{tr}}}^{2}M\in {\mathbb{C}}\backslash \mathrm{[0,\; 4]}$ ^17^. Let us now consider a superposition of mutually orthogonal elliptic (conservative spin dynamics in real magnetic field, see Fig. 1a) and hyperbolic (spin saturation in the direction of imaginary magnetic field, see Fig. 1b) transformations. For the Möbius transformation (14) this corresponds to *h*_*x*_ ≠ 0, *h*_*y*_ = 0, and *β* ≠ 0. Depending on the ratio $\varepsilon \equiv |\beta /{h}_{x}|$, the transformation (14) can be elliptic (*ε* < 1), loxodromic (*ε* > 1) or parabolic (*ε* = 1). As *ε* approaches 1 from below, the two fixed points of the elliptic transformation, which describes a steady state spin dynamics, move toward one another (see Fig. 2a) until they eventually coalesce into the single fixed point of a parabolic transformation at *ε* = 1, as shown in Fig. 2b. As *ε* is increased further, the fixed point splits into the attractive and repulsive centers of the hyperbolic transformation (see Fig. 2c), which corresponds to exponentially fast saturation of spin. The described transition plays an important role in spin dynamics: it is associated with the transition between regimes of unbroken and broken ${\mathscr{P}}{\mathscr{T}}$ symmetry^10^.

Expectation values of the spin Hamiltonian (1) evaluated at the fixed points, Eq. (12), ${E}_{\mathrm{1,2}}=\pm \sqrt{{h}_{x}^{2}+{\tilde{h}}_{y}^{2}}$, are directly related to the eigenvalues of the corresponding Möbius transformation matrix, Eq. (12), ${\lambda }_{\mathrm{1,2}}={{\rm{e}}}^{i\frac{{E}_{\mathrm{1,2}}t}{2}}$. They fully determine the types of the fixed points and the type of the transformation. The standard classification^17^ uses multipliers of the transformation

15 $${\kappa }_{\mathrm{1,2}}\equiv {\lambda }_{\mathrm{1,2}}^{-2}={{\rm{e}}}^{-i{E}_{\mathrm{1,2}}t},$$

such that |*κ*_1,2_| = 1 $({\kappa }_{\mathrm{1,2}}={{\rm{e}}}^{\pm i\theta }\ne 1)$ for elliptic transformations, *κ*_1,2_ = 1 for parabolic transformations, and *κ*_1,2_ ≠ 1 for loxodromic transformations (with real *κ*_1,2_ ≠ 1 in the special case of hyperbolic transformations). In the language of classical spin dynamics, this outcome fully accords with the above considerations.

## Conclusions

We have shown that the time evolution of linear classical single-spin systems has a simple interpretation in terms of Möbius transformations of ${{\mathbb{C}}}^{2}$, provided magnetic anisotropies are negligibly small. The ${\mathscr{P}}{\mathscr{T}}$ symmetry-breaking phase transition in such systems can be identified as a transition between elliptic and hyperbolic (via parabolic) classes of Möbius transformations appearing as solutions of the corresponding spin dynamics equations in complex stereographic coordinates. The established correspondence between linear spin dynamics and Möbius transformations reveals that any Möbius transformation can be produced by a unique superposition of an elliptic and hyperbolic transformations, corresponding to real and imaginary applied magnetic fields, respectively. We have demonstrated that the nonlinear LLGS equation describing dissipative STT-driven dynamics of a linear single-spin system can be written in a linear form, illustrating that such dynamics cannot produce any nonlinear effects, e.g. chaotic dynamic, for which additional time-dependent perturbation are necessary^11, 12^. The nonconservative effect of Slonczewski STT on spin systems, equivalent to the action of *imaginary* magnetic field, promises a unique tool for studying Lee-Yang zeros^18^ in ferromagnetic Ising and Heisenberg models.
